# Conduction treatment of temporal lobe epilepsy in rats: the dose-effect relationship between current resistance and therapeutic effect

**DOI:** 10.3389/fneur.2023.1181953

**Published:** 2023-05-25

**Authors:** Shaohui Zhang, Liu Yuan, Chunxiu Liu, Suhui Kuang, Jiaqi Wang, Shuli Liang, Ming Cong

**Affiliations:** ^1^Department of Neurosurgery, The First Affiliated Hospital of Jinzhou Medical University, Jinzhou, China; ^2^Functional Neurosurgery Department, National Children’s Health Center of China, Beijing Children’s Hospital, Capital Medical University, Beijing, China; ^3^Department of Neurosurgery, Chinese PLA General Hospital, Beijing, China; ^4^The State Key Laboratory of Transducer Technology, Aerospace Information Research Institute (AIR), Chinese Academy of Sciences, Beijing, China; ^5^Key Laboratory of Major Diseases in Children, Ministry of Education, Beijing, China

**Keywords:** temporal lobe epilepsy (TLE), current conduction, glutamate, interleukin 1β (IL-1β), high mobility group protein B1 (HMGB-1)

## Abstract

**Objective:**

To investigate the effect of current resistance on therapeutic outcomes, and the mechanism of current conduction treatment in a rat model of temporal lobe epilepsy (TLE).

**Methods:**

Rats were randomly divided into four groups: normal control, epileptic group, low-resistance conduction (LRC) and high-resistance conduction (HRC) group. The content of glutamate (Glu) and gamma-amino butyric acid (GABA) in the hippocampus was determined using a neurotransmitter analyzer. mRNA and protein expression of interleukin 1β (IL-1β) /IL-1 receptor 1(IL-1R1) and high mobility group protein B1 (HMGB-1)/toll-like receptor-4 (TLR-4) in hippocampal neurons were tested. Video electroencephalogram monitoring was used to record seizures and EEG discharges. Cognitive function in the rats was tested using the Morris water maze.

**Results:**

Glu/GABA ratio in the epileptic control and HRC groups was significant differences from LRC group. The levels of HMGB1/TLR4 and IL-1β/IL-1R1 in the LRC group and normal control group were significantly lower than those in epileptic control group (*p* < 0.01) and the HRC group. The mRNA levels of HMGB1/TLR4 and IL-1β/IL-1R1 in the LRC group and normal control group were significantly lower than those in epileptic control group. The frequency of total and propagated seizures was lower in the LRC group than in the epileptic control and HRC groups (*p* < 0.01). The numbers of platform crossings in the LRC group and normal control group were significantly higher than those in the epileptic control and HRC groups in the space exploration experiment.

**Conclusion:**

Current resistance affected seizure control and cognitive protection in rats with TLE treated by current conduction. The lower current resistance, the better seizure control and cognitive protection in rats with TLE treated by current conduction. Glu/GABA, IL-1β/IL-1R1, and HMGB1/TLR-4 may participate in the anti-seizure mechanism of current conduction treatment.

## 1. Introduction

Epilepsy is a common neurological disorder, affecting 8% of the population and approximately 70 million individuals worldwide (1, 2). Current therapeutic approaches for epilepsy consist of anti-seizure medications (ASMs), craniotomy, radiotherapy, and neuromodulation, which achieve noticeable clinical decreases in seizure burden. However, seizure control remains unsatisfactory, and approximately 40% of patients with epilepsy experience drug-resistant seizures or intolerance to ASM-related side effects (3, 4). Radiotherapy is seldom used in clinical treatment, and seizure control has varied significantly across different studies. Approximately 40% of patients experience continuous postoperative seizures after resective surgery (5, 6). The percentage of postoperative seizure freedom is only 10% in patients who have undergone alleviation surgery or neuromodulation due to a nonlocalizable epileptogenic zone or an epileptogenic zone involving the eloquent area (7–10). Therefore, it is necessary to explore novel treatments for drug-resistant epilepsy.

The basic concept underlying the mechanism of current treatment(s), including ASMs and epileptic surgery, is to inhibit the formation of neuronal super-synchronous discharges or to block the propagation of epileptiform discharges in the epileptic network (11, 12). Intracranial electroencephalogram (EEG) examination usually reveals a focal and fixed epileptogenic zone in patients, and focal high-voltage activity in the epileptogenic zone during the ictal period, which is always localized in the focal region for several seconds and has a long potential period before propagation. Furthermore, it is well known that current can flow from a higher-voltage region to a lower-voltage area. Therefore, our research team proposed a new concept of conduction of electrical current from a higher voltage area (the epileptogenic zone) to a lower voltage area (an extracranial region) to prevent the recruitment and propagation of epileptiform discharges (13). Current conduction has been confirmed to be feasible, and can decrease subclinical seizure on EEG and clinical seizure, especially propagated and generalized seizure, in a Sprague–Dawley (S–D) rat model of kainic acid-induced temporal lobe epilepsy (TLE). Furthermore, it can reduce apoptosis of hippocampal neurons (14) and, moreover, has been tested in a previous study (15).

Nevertheless, many questions still need research, such as whether conduction using various current levels can result in different therapeutic outcomes. The mechanisms of current conduction treatment involve amino acid transmitters and inflammatory factors, especially glutamate (Glu), gamma-amino butyric acid (GABA), interleukin (IL)-1β, IL-1 receptor (IL-1R), high mobility group protein B1 (HMGB-1), and toll-like receptor 4 (TLR-4), which can affect the electrical activity of neurons and glial cells and aggravate nerve cell damage (16–20).

Accordingly, we designed this study to examine the effects of epileptiform discharge to a source with near-zero voltage using different currents by adding resistance to examine the relationship between current level during conduction and seizure control and the mechanism of treatment.

## 2. Materials and methods

### 2.1. Animals and model

Two hundred healthy male S–D rats weighing 200–300 g were acquired from a commercial supplier (SPF Biotechnology Co., Ltd., Beijing, China). The animals were housed under the following conditions: temperature, 18°C–26°C; humidity, 40%–70%; and a cycle of 12 h dark and 12 h light.

The experimental rats were anesthetized by intraperitoneal injection of 1% pentobarbital sodium (40 mg/kg) and fixed using a stereotactic apparatus. The right hippocampal CA1 and CA3 regions were located using the Paxino stereotaxic brain map (13). Catheters were placed in the CA1 and CA3 areas and fixed to the skull. The conduction electrode was implanted through the catheter in the CA1 area and connected to the end of the electrode in a socket outside the scalp. One week later, 0.8 μg of kainic acid was slowly injected into the reserve catheter in the CA3 area using a microinjection pump under anesthesia. The rats were allowed to awaken naturally in an incubator. Establishment of model animals was considered to be successful when the rats exhibited seizures graded as Racine IV–V. Rats exhibiting seizures were randomly divided into three groups: epileptic control, low-resistance conduction, and high-resistance conduction group. Rats in the epileptic control group were not further treated. For rats in the low-resistance group, the conduction electrodes were connected to a source with near zero (±1 mV alternating current power supply) through the socket outside the scalp. For rats in the high-resistance group, the conduction electrodes were connected to a resistance of 10 KΩ and where the source was near zero (Supplementary Figure S1).

The rats in normal control were healthy S–D rats without kainic acid injection, anesthesia, and conduction electrodes or microdialysis electrode implantation.

### 2.2. Measurement of Glu/GABA transmitter levels

Glu and GABA levels were tested using a FAST16 mk-III neurotransmitter tester. A microdialysis electrode was calibrated for all experiments. The electrode was then implanted *via* a reserve catheter in the CA3 area after 7 days of current conduction. Calibrated parameters and corresponding calibration slopes were selected. Glu and GABA concentration was measured after baseline levels stabilized during recording.

### 2.3. Real-time polymerase chain reaction

The rats were euthanized or anesthetized after 24 h or 7 days of conduction treatment, respectively. The brains were removed, and the hippocampus was separated on an ice plate (Supplementary Figure S1), powdered, and total RNA was extracted using a commercially available total RNA extraction kit (TRIzol, Tiangen Biotech, Beijing, China; Catalog No. DP405-02). Experiments were performed in accordance with manufacturer’s instructions and as described in previous studies (21). RNA concentration and purity were measured using the ultraviolet absorption method and a spectrophotometer (Nanodrop Nd-2000, ThermoFisher Scientific, Waltham, MA, United States). Before measurement, the samples were zeroed with DEPC-treated water to dissolve RNA. Denaturing agarose gel electrophoresis was performed to determine the purity and degree of RNA degradation. Complementary DNA reverse transcription was performed using a commercially available kit (PrimeScript RT) with a gDNA eraser (Takara Bio., Shiga, Japan; Catalog No. RR047B) in accordance with manufacturer’s instructions. Finally, the target genes and internal reference for each sample were subjected to real-time polymerase chain reaction (RT-PCR), with three wells used for each sample (i.e., triplicate). The data were analyzed using the 2^−ΔΔCT^ method. The content of each type of mRNA in the first specimen of rat in normal control group was defined as 1 (i.e., reference), and the relative level of each type of mRNA in hippocampus was calculated for each rat. The primer sequences were list in Supplementary Table S1.

### 2.4. Western blot

Rats were euthanized or anesthetized after 24 h or 7 days of conduction treatment, respectively. The brains were removed and the hippocampus was separated on an ice plate and washed in precooled phosphate-buffered saline (0.02 mol/L, pH 7.0–7.2) to remove blood. RIPA protein extraction reagent (Huaxing Bochuang, China) was used to extract total protein. β-Actin (Sigma-Aldrich, St. Louis, MO, United States) was used as the internal reference (Supplementary Figure S2). Protein was quantified using a commercially available BCA kit (Pulilex, China) in accordance with manufacturer’s instructions.

### 2.5. Immunohistochemistry

Paraffin tissue sections were dewaxed in water, and were placed in a repair box filled with citric acid antigen repair buffer (pH 6.0) for antigen repair in a microwave oven. Endogenous peroxidase was blocked, and the tissue was sealed at room temperature for 30 min. After adding primary antibody, the tissue was incubated overnight at 4°C, and secondary antibody was added the next day. After the sections were slightly dried, freshly prepared DAB coloration solution was added. The coloration time was controlled under a microscope until the positive color was brownish yellow. The sections were rinsed with tap water to stop the coloration reaction. Nuclei were stained with hematoxylin, and microscopic examination, image collection, and analysis were performed after dehydration.

### 2.6. Immunofluorescence

Paraffin slices were dewaxed in water, and placed in a repair box filled with EDTA antigen repair buffer (pH 8.0) for antigen retrieval in a microwave oven. After the slices were dried slightly, a circle was drawn around the tissue using a histochemical pen. A spontaneous fluorescence-quenching agent was added to the circle for 5 min and then rinsed with running water for 10 min. Bovine serum albumin was dropped into the circle and the slices were incubated for 30 min. After adding primary antibody, the tissue was incubated overnight at 4°C, and secondary antibody was added the next day. The nucleus was counterstained using DAPI staining solution. After the film was sealed, the sections were observed under a fluorescence microscope for image collection.

### 2.7. Video-EEG monitoring

The recording electrode was implanted *via* a reserve catheter placed in the right CA3 area, and another needle electrode was implanted in the left CA3 area by drilling a hole in TLE model rats. Video EEG and epileptic seizures were recorded using a 32-lead video EEG recorder (Nicolet, Natus Neurology Inc., Middleton, WI, United States) from 09:00 to 14:00 every day. EEG seizures were recognized against background by their large amplitude (>3 times baseline amplitude) and high-frequency EEG activity (≥5 Hz) for at least 2 s (13). Epileptic seizures and EEG changes were observed within 3 days of current conduction treatment. Two EEG experts reviewed the video results.

### 2.8. Morris water maze

The Morris water maze experiment was performed as described in a previous study (22) and used to assess the following two skills (Supplementary Figure S3).

#### 2.8.1. Positioning navigation

The duration of this experiment was 5 days. The rats were placed into the water at four water entry points facing the pool wall once per day, and escape latency (the time to when the rats found the platform under the water) was recorded. If the platform was not found within 2 min, the operator placed the rat on the platform to rest for approximately 30 s and recorded the latency as 2 min. The average period was determined as the mean latency on days 3, 4, and 5.

#### 2.8.2. Space exploration

On the second day after training, the platform hidden in the water was removed, and the rats were placed into the pool at one entry point, and the number of times the rats crossed where the platform was located within 120 s was recorded.

### 2.9. Statistical analysis

Statistical analyses were performed using SPSS version 20.0 (IBM Corporation, Armonk, NY, United States). Outcomes are expressed as percentage, mean, and standard deviation (SD). Chi-squared and Fisher’s exact tests were used to analyze categorical variables. The *t* and *f*-tests were used to compare continuous variables. Analysis of variance (ANOVA) and Kruskal–Wallis tests were used for multiple comparisons. Statistical significance was defined as a two-tailed error probability <0.05 (i.e., *p* < 0.05).

## 3. Results

### 3.1. Glu/GABA transmitter levels

Glu and GABA levels were tested in five rats in three experimental groups after 7 days of current conduction treatment. There were no significant differences in Glu and GABA levels or in the Glu/GABA ratio between the epileptic control and high-resistance conduction groups. However, significant differences were found in Glu level at 40 min, 80–180 min, GABA content at 180 min and 20–140 min, and Glu/GABA ratio at 20–180 min between the epileptic control and low-resistance conduction groups. Furthermore, significant differences were also found in GABA level at 120, 140, 180 and 20–80 min, and the Glu/GABA ratio at 20–120 min between the high-resistance and low-resistance conduction groups (Table 1).

**Table 1:** Glutamate (Glu) and gamma-amino butyric acid (GABA) level and Glu/GABA ratio in rat hippocampus in three experimental groups.

	−40 min	−20 min	0 min	20 min	40 min	60 min	80 min	100 min	120 min	140 min	160 min	180 min
Epileptic control group												
Glu	100.33 ± 0.26	97.66 ± 4.51	100.97 ± 1.64	120.09 ± 8.09	128.10 ± 7.78	130.93 ± 12.45	140.16 ± 16.30	144.94 ± 27.82	150.33 ± 38.33	150.13 ± 35.64	139.67 ± 24.21	144.44 ± 30.78
GABA	101.22 ± 2.38	100.55 ± 2.70	101.80 ± 1.44	55.63 ± 14.52	56.05 ± 7.78	56.06 ± 4.89	47.50 ± 10.15	57.17 ± 11.99	49.92 ± 9.60	61.44 ± 10.38	59.11 ± 8.81	54.86 ± 10.55
Glu/GABA	0.99 ± 0.02	0.97 ± 0.05	0.99 ± 0.02	2.31 ± 0.75	2.32 ± 0.25	2.34 ± 0.25	2.52 ± 0.66	2.60 ± 0.60	3.07 ± 0.86	2.54 ± 0.88	2.44 ± 0.72	2.74 ± 0.88
High-resistance conduction group												
Glu	100.28 ± 2.42	99.52 ± 2.15	100.77 ± 1.95	116.56 ± 8.01	116.32 ± 12.22	129.08 ± 13.50	124.61 ± 20.10	123.09 ± 20.38	118.88 ± 13.53	116.39 ± 15.55^*^	115.44 ± 7.92^*^	119.64 ± 13.93
GABA	100.78 ± 0.96	87.89 ± 3.15	102.47 ± 6.50	63.38 ± 17.68	73.70 ± 21.56	70.41 ± 12.26^*^	67.42 ± 12.16^*^	72.62 ± 27.10	66.15 ± 14.56	70.54 ± 16.68	77.43 ± 22.55	73.89 ± 21.44
Glu/GABA	1.00 ± 0.02	1.02 ± 0.05	0.99 ± 0.07	1.96 ± 0.59	1.73 ± 0.68^*^	1.74 ± 0.39^**^	1.90 ± 0.45	1.84 ± 0.58^*^	1.87 ± 0.39^**^	1.73 ± 0.45^*^	1.57 ± 0.36^*^	1.72 ± 0.46^*^
Low-resistance conduction group												
Glu	101.36 ± 2.13	99.21 ± 2.53	100.74 ± 1.67	107.83 ± 5.27	112.38 ± 4.92^*^	114.96 ± 5.77	106.85 ± 7.79^**^	105.48 ± 7.90^*^	104.07 ± 4.44^**^	104.71 ± 8.47^**^	109.10 ± 8.97^**^	100.72 ± 3.85^**^
GABA	100.12 ± 3.8	100.49 ± 1.74	103.87 ± 6.55	101.04 ± 8.41^**,&&^	99.70 ± 10.28^**,&^	101.23 ± 10.21^**,&&^	102.23 ± 10.31^**,&&^	95.42 ± 12.59^**^	103.39 ± 13.35^**,&&^	113.84 ± 15.76^**,&&^	85.56 ± 16.98	102.70 ± 14.53^**,&^
Glu/GABA	1.01 ± 0.04	0.99 ± 0.02	0.97 ± 0.06	1.08 ± 0.12^**,&^	1.13 ± 0.09^**,&^	1.15 ± 0.16^**,&&^	1.06 ± 0.10^**,&^	1.13 ± 0.20^**,&^	1.02 ± 0.12^**,&^	0.94 ± 0.17^**^	1.32 ± 0.28^**^	1.00 ± 0.15^**^

^**^p < 0.01, ^*^p < 0.05 the data in this group compared to relative data in control group. ^&&^*p* < 0.01, ^&^*p* < 0.05 the data in this group compared to relative data in high-resistance conduction group.

### 3.2. mRNA levels of IL-1β/IL-1R1 and HMGB-1/TLR-4

**Figure 1:**
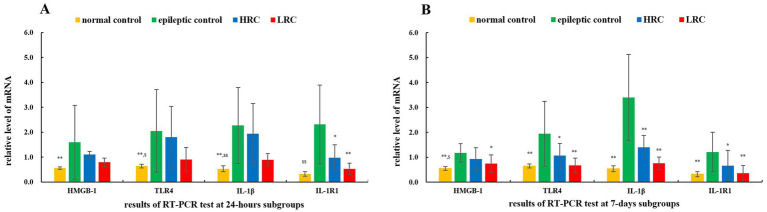
Relative mRNA level of interleukin (IL)-1β/IL-1R1 and high mobility group protein B1 (HMGB-1)/toll-like receptor (TLR)-4 at 24 h **(A)** and 7 days **(B)** after conduction. ^**^p < 0.01, ^*^p < 0.05，the data in this group compared to data in epileptic control group at the same time. ^$$^*p* < 0.01, ^$^*p* < 0.05, the data in this group compared to data in high-resistance conduction group at the same time. LRC, low-resistance conduction; HRC, high-resistance conduction. **(A)** Shows the mean mRNA levels of IL-1β/IL-1R1 and HMGB-1/TLR-4 in normal control group (*p* < 0.01), and IL-1β/IL-1R1 (*p* < 0.01) and TLR-4 (*p* < 0.05) in LRC group are significantly lower than those in the epileptic control group, also, the mRNA level of IL-1β1 (*p* < 0.01) and TLR-4 (*p* < 0.05) in normal control group, and IL-1β (*p* < 0.05) in LRC group are significantly lower than those in HRC groups at 24 h after current conduction treatment. **(B)** Shows the mean mRNA levels of IL-1β/IL-1R1 and HMGB-1/TLR-4 in normal control group (*p* < 0.01) and low-resistance conduction group, and IL-1β (*p* < 0.01), IL-1R1 and TLR-4 (*p* < 0.05) in HRC group were significantly lower than those in the epileptic control group, also, the mRNA level of HMGB-1 (*p* < 0.05) in normal control group was significantly lower than it in HRC group at 7 days after current conduction treatment.

After current conduction treatment for 24 h or 7 days, 12 and 9 rats completed the test of mRNA levels of IL-1β/IL-1R1 and HMGB-1/TLR-4 in each group, respectively. The level of mRNA in the normal control group was set as 1 (i.e., reference), and the relative data and statistical analysis results of the other samples are shown in Figure 1. The mean mRNA levels of IL-1β/IL-1R1 and HMGB-1/TLR-4 in normal control group (*p* < 0.01), and IL-1β/IL-1R1 (*p* < 0.01) and TLR-4 (*p* < 0.05) in low-resistance conduction group were significantly lower than those in the epileptic control group, also, the mRNA level of IL-1β1 (*p* < 0.01) and TLR-4 (*p* < 0.05) in normal control group, and IL-1β (*p* < 0.05) in low-resistance conduction group were significantly lower than those in high-resistance conduction group at 24 h after current conduction treatment. At 7 days after current conduction treatment, the mean mRNA levels of IL-1β/IL-1R1 and HMGB-1/TLR-4 in normal control group (*p* < 0.01) and low-resistance conduction group, and IL-1β (*p* < 0.01), IL-1R1 and TLR-4 (*p* < 0.05) in high-resistance conduction group were significantly lower than those in the epileptic control group, also, the mRNA level of HMGB-1 (*p* < 0.05) in normal control group was significantly lower than it in high-resistance conduction group (Figure 1).

### 3.3. Protein content of IL-1β/IL-1R1 and HMGB-1/TLR-4

**Figure 2:**
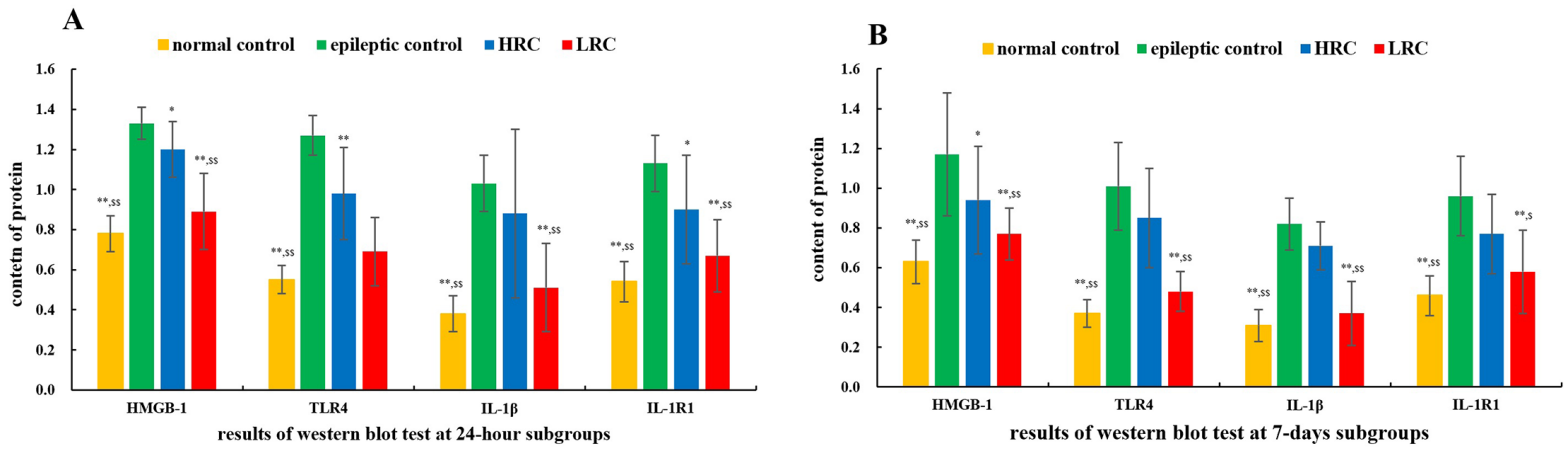
Comparison of protein levels among the different groups at 24 h **(A)** and 7 days **(B)** after conduction. ^**^p < 0.01, ^*^p < 0.05，the data in this group compared to data in epileptic control group at the same time. ^$$^*p* < 0.01, ^$^*p* < 0.05, the data in this group compared to data in HRC group at the same time. LRC, low-resistance conduction; HRC, high-resistance conduction. This figure shows the levels of IL-1β/IL-1R1 and HMGB-1/TLR-4 in normal control group and LRC group were significantly lower than those in the epileptic control group (*p* < 0.01) and HRC group in either the 24 h or the 7 days subgroup. The levels of HMGB-1, IL-1R1 (*p* < 0.05) and TLR-4 (*p* < 0.01) in HRC group were significantly lower than those in the epileptic control group at 24 h after current conduction treatment. The levels of HMGB-1 and IL-1R1 (*p* < 0.05) in HRC group were significantly lower than it in the epileptic control group at 7 days after current conduction treatment.

Protein levels of IL-1β/IL-1R1 and HMGB-1/TLR-4 were quantified using Western blot techniques. The results of the relative qualification of these proteins are shown in Figure 2. There were 18 samples in each group, including 9 in the 24 h subgroup and 9 in the 7 days subgroup. Either in the 24 h or 7 days subgroup, the levels of IL-1β/IL-1R1 and HMGB-1/TLR-4 in normal control group and low-resistance conduction group were significantly lower than those in the epileptic control group (*p* < 0.01) and high-resistance conduction group. The contents of HMGB-1, IL-1R1 (*p* < 0.05) and TLR-4 protein (*p* < 0.01) in high-resistance conduction group were significantly lower than those in the epileptic control group at 24 h after current conduction treatment. The contents of HMGB-1 and IL-1R1 protein (*p* < 0.05) in high-resistance conduction group were significantly lower than it in the epileptic control group at 7 days after current conduction treatment.

### 3.4. Cognitive function

Five rats in each group completed the Morris water maze experiment after 7 days of current conduction treatment. Significant difference was found in latency period of the positioning navigation experiment between normal control group and each experimental groups. However, the mean (±SD) number of times the rats crossed the platform was in normal control group (12.2 ± 3.83/120 s) (*p* < 0.01) the low-resistance conduction group (9.4 ± 1.34/120 s) (*p* < 0.05), which was significantly higher than in the epileptic control group (5.6 ± 2.07/120 s) and high-resistance conduction group (6.00 ± 1.58/120 s) (Supplementary Figure S4).

### 3.5. Video-EEG findings and clinical seizure

**Figure 3:**
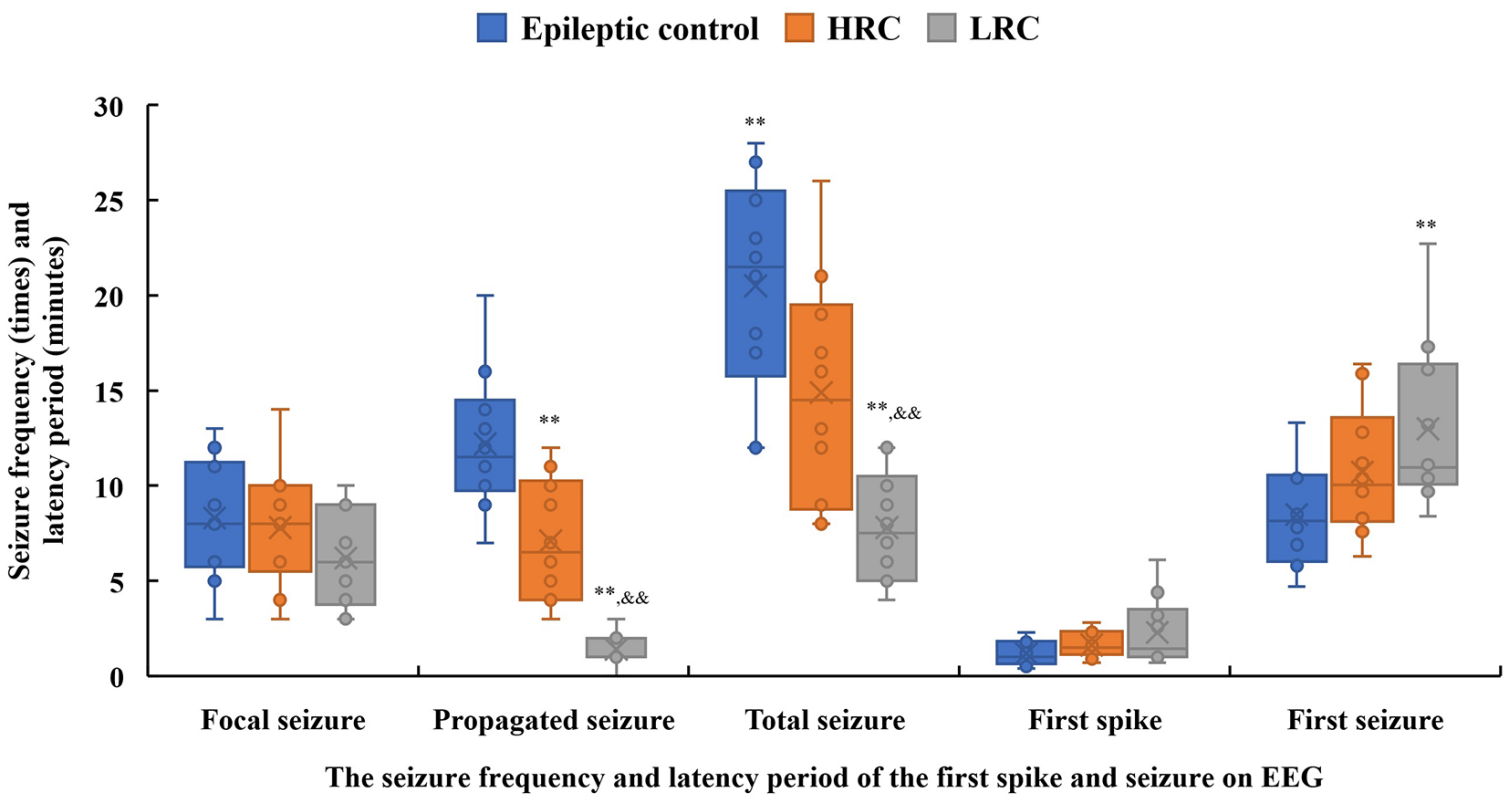
Seizure frequency (times, solid box) and latency period (minutes, hollow box) of the first spike and seizure on electroencephalogram recording in the three groups. ^**^p < 0.01, ^*^p < 0.05, compared to data in the control group; ^&&^*p* < 0.01, ^&^*p* < 0.05, compared to data in the high-resistance conduction group. This figure shows seizure frequency and latency period of the spike discharge and seizure on electroencephalogram. The latency period of the first seizure in the low-resistance conduction group was significantly longer than that in the control group (*p* < 0.01). Furthermore, the frequencies of total and propagated seizures were lower in the low-resistance conduction group than in the control and high-resistance groups (*p* < 0.01), and the frequencies of total seizures (*p* < 0.05) and propagated seizures (*p* < 0.01) were lower in the high-resistance conduction group than in the epileptic control group.

EEG seizures and epileptiform discharges were recorded for all 10 rats in each experimental group (Supplementary Figure S5), and none EEG seizure or epileptiform discharge was found in normal control group. The earliest spike occurred 0.4 min after the EEG electrode was connected, and no significant difference was found in latency period of the first spike among the three experimental groups. The first EEG seizure was recorded at 4.7 min, and the latency period of the first seizure in the low-resistance conduction group was significantly longer than that in the epileptic control group (*p* < 0.01) (Figure 3). Furthermore, the frequencies of total and propagated seizures were lower in the low-resistance conduction group than those in the epileptic control group and high-resistance groups (*p* < 0.01), and the frequency of total seizures (*p* < 0.05) and propagated seizures (*p* < 0.01) were lower in the high-resistance conduction group than that in the epileptic control group. However, there was no significant difference in the frequency of focal seizures among the experience groups (Figure 3).

All 10 rats in each experimental group exhibited clinical seizures, and none seizure was found in normal control group. The seizure frequency on the first day was significantly higher than that on the second or third day in all experimental groups (*p* < 0.01), and the seizure frequencies on the second day were significantly higher than those on the third day in the two conduction treatment groups (*p* < 0.01). The total seizure frequency and seizure frequency on each day were significantly lower in the low-resistance conduction group than them in the epileptic control group. The total seizure frequency and seizure frequency on the first day of low-resistance conduction treatment were also significantly lower than those in the high-resistance conduction group. In addition, the total seizure frequency and seizure frequency on the first and third days of high-resistance conduction were significantly lower than those in the epileptic control group (Table 2).

**Table 2:** Clinical seizure times in the first 3 days in all experience groups.

Group	First day	Second day	Third day	Total
Epileptic control group (n = 10)	22.40 ± 11.24	4.20 ± 2.04^&&^	2.80 ± 2.04^&&^	29.40 ± 11.02
High-resistance conduction group (n = 10)	13.70 ± 5.38^*^	3.40 ± 1.58 ^&&^	1.20 ± 0.92^*,&&,##^	18.30 ± 6.13^**^
Low-resistance conduction group (n = 10)	6.70 ± 5.40^**,$$^	2.30 ± 0.95^*,&&^	0.50 ± 0.85^**,&&,##^	9.50 ± 6.36^**,$^

^**^p < 0.01, ^*^p < 0.05, the data in this group compared with the data in epileptic control group at the same time. ^$$^*p* < 0.01, ^$^*p* < 0.05, the data in this group compared with the data in high-resistance conduction group at the same time. ^&&^*p* < 0.01, ^&^*p* < 0.05, the data in this day compared with the data in first day in same group. ^##^*p* < 0.01, ^#^*p* < 0.05, the data in this day compared with the data in second day in same group.

### 3.6. Immunohistochemistry

**Figure 4:**
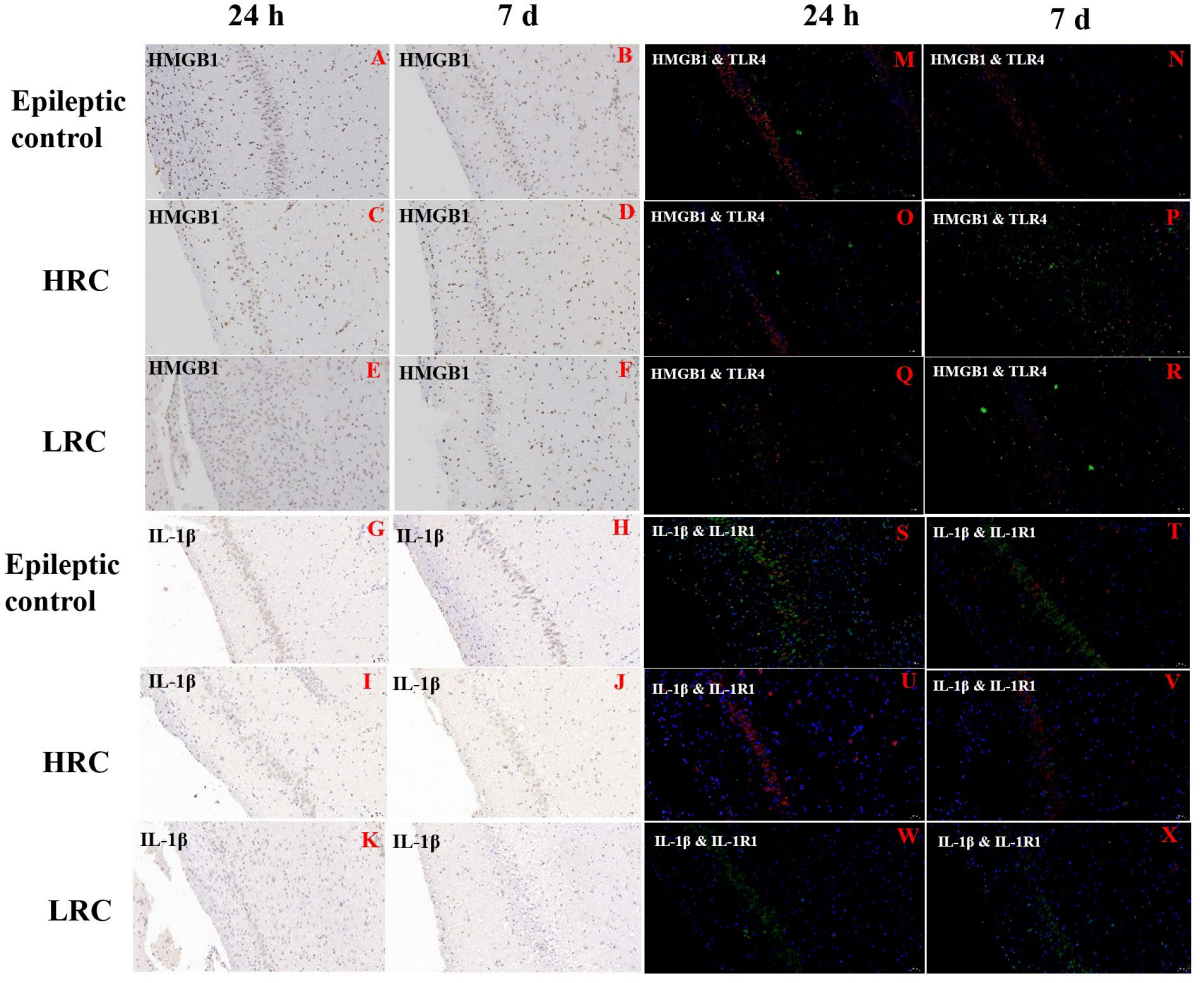
Immunohistochemistry of HMGB-1 **(A–F)** and IL-1β **(G–L)** and immunofluorescence of HMGB-1/TLR-4 **(M–R)** and IL-1β/IL1-R1 **(S–X)** in hippocampus (original magnification, ×20). Positive expression of IL-1β and HMGB1 is green, and positive expression of IL1-R1 and TLR4 is red in the immunofluorescence images. This figure shows the partial results of immunofluorescence and immunohistochemistry. The percentage of HMGB-1 positive cells was lower in the low-resistance conduction group **(E,F)** than in the epileptic control **(A,B)** and high-resistance conduction groups **(C,D)**, and the percentage of IL-1β positive cells was lower in the low-resistance conduction group **(K,L)** than in the epileptic control **(G,H)** and high-resistance conduction groups **(I,J)** at either 1 or 7 days after conduction treatment. The positive expression of HMGB-1/TLR-4 were lower in low-resistance conduction group **(Q,R)** than in the high-resistance conduction group **(O,P)** and epileptic control group **(M,N)** in the hippocampus. Also, the positive expression of IL1-R1/IL-1β were lower in low-resistance conduction group **(W,X)** than in the epileptic control group **(S,T)** in the hippocampus while the positive expression of IL1-R1 in the high-resistance conduction group **(U,V)** was increased compared to the epileptic control group **(S,T)**.

The nucleus appeared blue with hematoxylin staining, and positive cytoplasm with proteins IL-1β, IL1-R1, HMGB1, and TLR4 appeared tan in color with DAB staining. The percentages of positive cells were lower in low-resistance conduction group than them in the epileptic control and high-resistance conduction groups at either 1 or 7 days after conduction treatment (Figures 4A–L).

### 3.7. Immunofluorescence

The nucleus stained with DAPI appeared blue under the excitation of ultraviolet light; positive expression of IL-1β and HMGB1 is green, and positive expression of IL1-R1 and TLR4 is red, marked by the corresponding fluorescein tag. The percentages of cells positive for IL-1β, IL1-R1, HMGB1, and TLR4 were lower in the low-resistance conduction group than them in the epileptic control group and high-resistance conduction group at either 1 or 7 days after conduction therapy. Furthermore, the percentages of cells positive for IL-1β, IL1-R1, HMGB1, and TLR4 were slightly lower in the high-resistance conduction group than those in the epileptic control group (Figures 4M–X).

## 4. Discussion

The foundational concept of electrical conduction therapy is different from that of traditional resection surgery or neuromodulation. The basic concept of electric conduction therapy is to divert current from the epileptic focus to the outside of the brain, thereby reducing the spread of epileptic discharge and the occurrence of seizures. In a previous study involving rats with TLE, we confirmed the effectiveness and feasibility of electrical conduction therapy. Therefore, the present investigation examined the influence of varying resistance on the effects of current conduction. At the same time, we further investigated its therapeutic mechanism by examining amino acid transmitters and inflammatory factors in a rat model of TLE.

EEG monitoring and video recording of seizures were performed in the three groups within 3 days of conduction treatment of S–D rats with TLE. The time from the beginning of the recording to the first seizure was significantly longer in the low-resistance conduction group than in the epileptic control group. The total number of EEG seizures and the number of propagated EEG seizures in the low-resistance conduction group were significantly lower than those in the high-resistance conduction and epileptic control groups, and the high-resistance conduction group was significantly lower than the epileptic control group. The total number of clinical seizures was significantly lower in the low-resistance than in the high-resistance conduction and epileptic control groups, and significantly lower in the high-resistance conduction group than in the epileptic control group. The results confirmed that the number of clinical seizures and EEG epileptiform discharges in rats with TLE were significantly reduced by current conduction treatment, with lower current resistance and better seizure control.

In this study, the Morris water maze was used to test cognitive function in rats with TLE. There was no statistical difference in latency of the positioning navigation experiment among the three groups of rats after 7 days of current conduction treatment, while the number of platform crossings in the low-resistance conduction group was significantly higher than that in the epileptic control and high-resistance conduction groups in the space exploration experiment. These results suggest that low-resistance—but not high-resistance—conduction treatment could protect cognitive function in rats with TLE.

Microdialysis, a neurochemical technology used to monitor dynamic concentration changes in extracellular neurotransmitters, has been used to measure changes in excitatory and inhibitory neurotransmitter concentrations in animals exhibiting seizures (23, 24). Epilepsy is related to the imbalance of Glu and GABA, and changes in Glu and GABA play an important role in the formation of epileptic networks and the initiation and transmission of spontaneous seizures (25, 26). In this study, Glu and GABA concentrations in the hippocampus revealed that the Glu/GABA ratio in the low-resistance conduction group was significantly lower than that in the epileptic control group and high-resistance conduction groups, and the Glu/GABA ratio in the high-resistance conduction group was also significantly lower than that in the epileptic control group. Current conduction intervention significantly decreased the Glu/GABA ratio in the hippocampus, and seizure attack frequency was also reduced with a decrease in the Glu/GABA ratio and conduction resistance.

The neuroimmune inflammatory response mediated by IL-1β/IL-1R1 and HMGB1/TLR4 signaling pathways plays an important role in the pathogenesis of epilepsy (27). These two signaling pathways are biomarkers for prediction, prognosis, and targeted therapy for epilepsy (28). In this study, we analyzed these factors in the hippocampus of rats with TLE treated with current conduction for 24 h and for 7 days. The gene and protein expression of IL-1β/IL-1R1 and HMGB1/TLR-4 after low-resistance conduction treatment were significantly lower than those in the epileptic control group. IL-1β/IL-1R1 and HMGB1/TLR-4 may participate in the anti-seizure effect of current conduction treatment.

The present study had some limitations. First, the conduction electric current was not recorded directly. Second, conduction was not always continuous in all rats because near-zero power could not be implanted subcutaneously. Third, the sample size of rats and therapeutic period were limited.

In conclusion, this study evaluated the relationship between current resistance, seizure control, and cognitive protection in rats with TLE treated with current conduction. The lower current resistance, the better seizure control and cognitive protection in rats with TLE treated by current conduction. Glu/GABA, IL-1 β/IL-1R1, and HMGB1/TLR-4 may participate in the anti-seizure mechanism of current conduction treatment.

## Data availability statement

The raw data supporting the conclusions of this article will be made available by the authors, without undue reservation.

## Ethics statement

The animal study was reviewed and approved by Animal Ethics Committee of SPF Biotechnology Co., Ltd.

## Author contributions

SZ: conceptualization, acquisition and analysis of data, writing— original draft, and writing—review & editing. LY: acquisition of data, analysis and interpretation of data, writing—original draft, and writing— review & editing. CL: design of the study, investigation, and writing— review & editing. SK and JW: analysis and interpretation of data and writing—review & editing. SL: conceptualization, design of the study, funding acquisition, investigation, writing—original draft, and writing— review & editing. MC: conceptualization, design of the study, investigation, writing—original draft, and writing—review & editing. All authors contributed to the article and approved the submitted version.

## Funding

This study was funded by National Nature Science Foundation of China (81771388, 82071448, SL). The funder was not involved in the study design, data collection and analysis, interpretation of data and the writing of the report.

## Conflict of interest

The authors declare that the research was conducted in the absence of any commercial or financial relationships that could be construed as a potential conflict of interest.

The handling editor TL declared a shared affiliation with the author(s) LY, SK, JW, and SL at the time of review.

## Publisher’s note

All claims expressed in this article are solely those of the authors and do not necessarily represent those of their affiliated organizations, or those of the publisher, the editors and the reviewers. Any product that may be evaluated in this article, or claim that may be made by its manufacturer, is not guaranteed or endorsed by the publisher.
