# Supplementary material for: Conduction treatment of temporal lobe epilepsy in rats: the dose-effect relationship between current resistance and therapeutic effect
Source: Front Neurol. 2023 May 25;14:1181953. doi: 10.3389/fneur.2023.1181953 (PMC10248515; doi:10.3389/fneur.2023.1181953)
Supplement: Supplementary file 1 [file Data_Sheet_1.PDF]

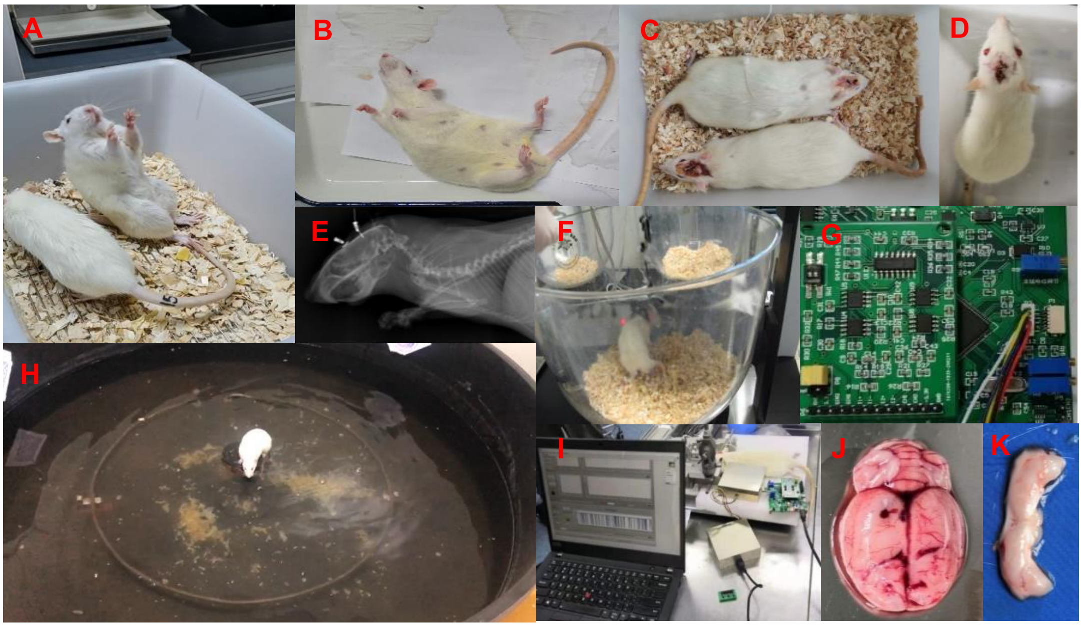

Supplementary Figure 1. The picture of experience course, including build rat model (A, B), conduction electrodes implantation (C-E), content of amin acid testing (F), electric board for power with near zero voltage (G), morris water maze test (H)m current conduction test (I), whole brain (J), and hippocampus sample (K).

Supplementary Table 1. The primer sequences

Prime		Sequences (5'to3')	Product size（bp）
**IL1R1**	Forward	AAGTGGAATGGGTCGGAAAT	156
**IL1R1**	Reverse	GCAGATGAACGGATAGCGATA	
**HMGB-1**	Forward	GGATGACAAGCAGCCCTATG	119
**HMGB-1**	Reverse	CCTTGACCACCCCCTTTT	
**TLR4**	Forward	AAGACTATCATCAGTGTATCGGTGG	181
**TLR4**	Reverse	CGTTTCTCACCCAGTCCTCATT	
**IL1β**	Forward	GGGATGATGACGACCTGCTA	150
**IL1β**	Reverse	CCACTTGTTGGCTTATGTTCTG	
**NMDAR**	Forward	GCTCCTGCAACCCTCACTT	106
**NMDAR**	Reverse	GCTTGTAGGCGATCTCAATGAA	
**ACTIN**	Forward	CCATCTACGAGGGCTATGCT	150
**ACTIN**	Reverse	CTTTGATGTCACGCACGATT	

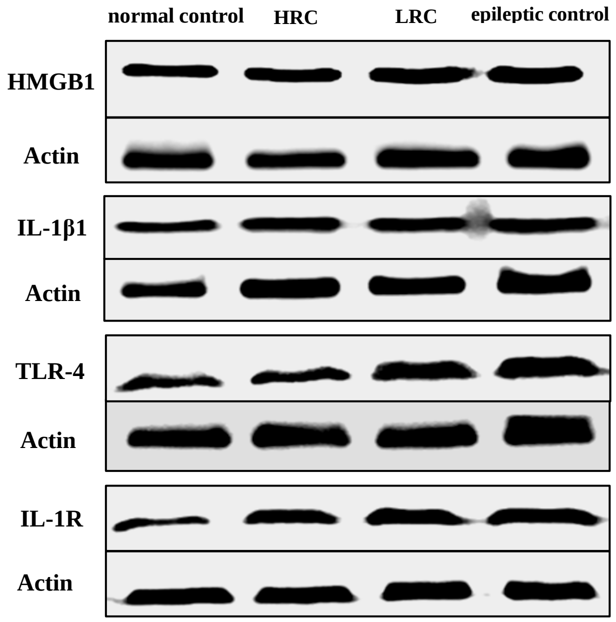

Supplementary Figure 2

The electrophoresis picture of high mobility group protein B1 (HMGB-1)/toll-like receptor (TLR)-4 and interleukin (IL)-1β/IL-1R1 proteins in the 7-day subgroup. HRC: high-resistance conduction, LRC: low--resistance conduction.

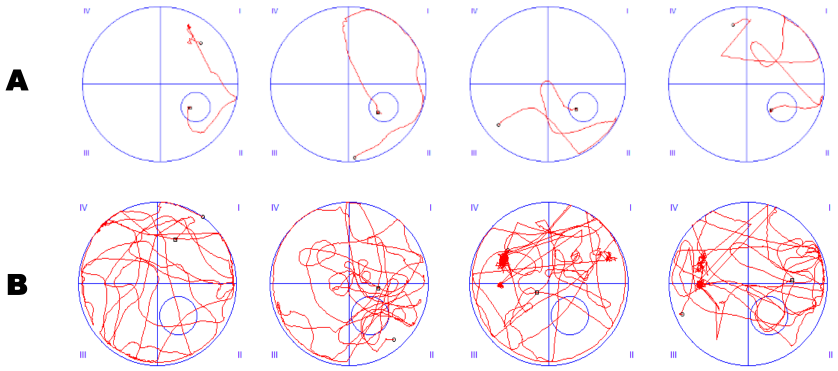

Supplementary Figure 3 Example of Morris water maze experiment results. Figure A: trace plot of once positioning navigation. Figure B: trace plot of space exploration in 120 seconds.

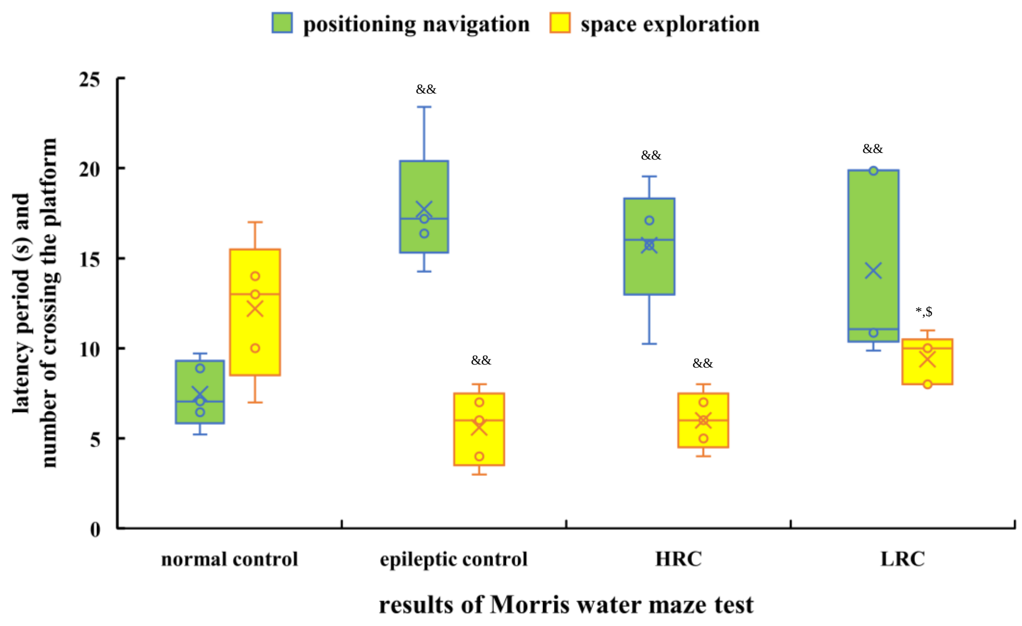

Supplementary Figure 4. Latency period of positioning navigation experiment and number of times the rats crossed the platform in four groups. HRC: high-resistance conduction, LRC: low--resistance conduction. *P<0.05，the data in this group compared to data in epileptic control group at the same time; $P<0.05, the data in this group compared to data in high-resistance conduction group at the same time; && P<0.01, the data in this group compared to data in normal control group.

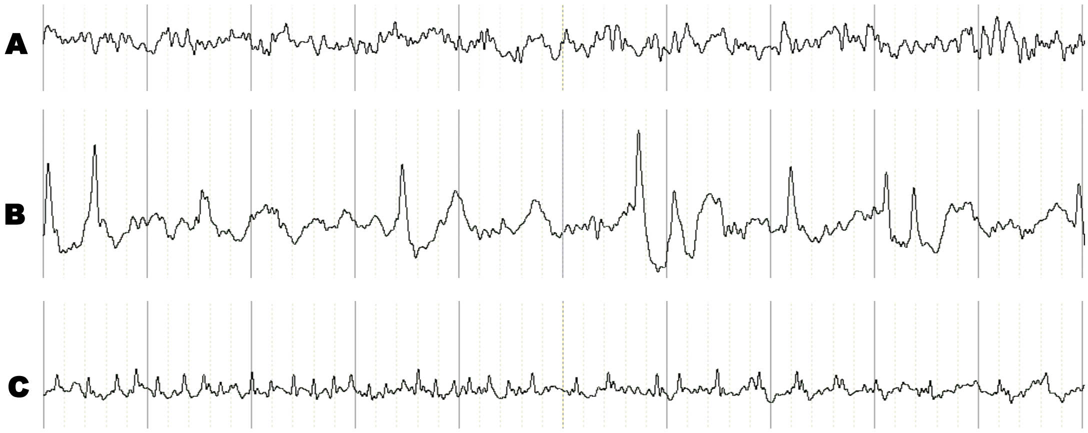

Supplementary Figure 5. EEG finding in rat model with temporal lobe epilepsy. A: background activity. B: spike and spike-slow wave with high attitude. C: EEG seizure with rhythmic discharges.
